# A cross-species transcriptomics approach to identify genes involved in leaf development

**DOI:** 10.1186/1471-2164-9-589

**Published:** 2008-12-05

**Authors:** Nathaniel Robert Street, Andreas Sjödin, Max Bylesjö, Petter Gustafsson, Johan Trygg, Stefan Jansson

**Affiliations:** 1 Umeå Plant Science Centre, Department of Plant Physiology, Umeå University, SE-901 87 Umeå, Sweden; 2 Research Group for Chemometrics, Department of Chemistry, Umeå University, SE-901 87 Umeå, Sweden

## Abstract

**Background:**

We have made use of publicly available gene expression data to identify transcription factors and transcriptional modules (regulons) associated with leaf development in *Populus*. Different tissue types were compared to identify genes informative in the discrimination of leaf and non-leaf tissues. Transcriptional modules within this set of genes were identified in a much wider set of microarray data collected from leaves in a number of developmental, biotic, abiotic and transgenic experiments.

**Results:**

Transcription factors that were over represented in leaf EST libraries and that were useful for discriminating leaves from other tissues were identified, revealing that the C2C2-YABBY, CCAAT-HAP3 and 5, MYB, and ZF-HD families are particularly important in leaves. The expression of transcriptional modules and transcription factors was examined across a number of experiments to select those that were particularly active during the early stages of leaf development. Two transcription factors were found to collocate to previously published Quantitative Trait Loci (QTL) for leaf length. We also found that miRNA family 396 may be important in the control of leaf development, with three members of the family collocating with clusters of leaf development QTL.

**Conclusion:**

This work provides a set of candidate genes involved in the control and processes of leaf development. This resource can be used for a wide variety of purposes such as informing the selection of candidate genes for association mapping or for the selection of targets for reverse genetics studies to further understanding of the genetic control of leaf size and shape.

## Background

Leaves are of fundamental importance to life on earth, representing the powerhouses of most food chains and are, ultimately, the source of energy that sustains humanity. Although much has been learnt about the biochemical and physiological functioning of leaf-level processes such as photosynthesis [1], still relatively little is known about why leaves are the size and shape they are and how they come to be so. There are genes known to affect meristomatic pattern formation (e.g. *AS1 *and *WUS*, *KNOX *and *CLV*, see [2] for a review of leaf development) and the rate of leaf primordia initiation [3] as well as genes that contribute to the determination of leaf length (*ROT3 *[4], *LNG *[5]) and width (*AN*, [6]), with less being known about the determination of leaf size (although [7] provide exciting insight). Although the two now classic examples of *ROT *and *AN *clearly affect leaf width and length, each will likely explain only a small proportion of the variation that exists for these traits, as numerous examples suggest that leaf size and shape are under complex polygenic control [8-17]. The vast majority of work identifying genes that function in the control of leaf size and shape has, to date, been conducted using the model species *Arabidopsis thaliana*, with most genes being identified through mutant screens, and, to the best of our knowledge, with no examples of evidence that such genes show variation between ecotypes of *Arabidopsis *with contrasting leaf shape or size. Beyond this, the work in [18] showed how conclusions drawn about gene function in *Arabidopsis*, which has simple leaves, may not hold true in species with more complex, dissected leaf forms.

We are interested in identifying genes that are functionally important in determining natural variation in leaf size, shape, and development. Forward genetic screens represent an important approach for identifying genes that have the potential to contribute to natural variation of phenotypic traits. However, such mutant screens are not directed by natural variation in phenotype and there is no reason to expect that genes identified through induced mutation (or other means of disrupting gene function) are those that have been acted upon by natural selection, which could render them interesting but unimportant in an ecological/evolutionary context. The opposite paradigm of reverse genetics offers a way of identifying functionally important loci but, in the case of QTL or association mapping, is time-consuming and often significantly limited by the biology of the system being studied – for example in forest tree species the generation of mapping populations is a very long-term commitment and the subsequent QTL (Quantitative Trait Loci) mapping resolution is of questionable value if the aim is to identify genes, or even causal polymorphisms, underlying a QTL (although association mapping largely overcomes this resolution limitation, for example see [19], but requires greater starting information to enable targeting of relevant loci). A third approach, which represents a refinement to a reverse genetics pipeline, is to identify genes whose expression pattern(s) suggest they might be functioning during a developmental process or response to stimuli/stress (i.e. functional genomics, or perhaps initially 'guilt-by-association') and to combine this information with QTL mapping, and approach that was termed 'genetical genomics' [20]. This proposed view of how to consider gene expression (or similarly peptide/metabolite abundance) in a genetical context is currently receiving considerable attention, extension and refinement of methodology [21-25]. Association mapping and genetical genomics both represent major motivations for the efficient identification of candidate genes, particularly where whole-genome assays cannot be utilised, such as in non-model systems or species such as aspens, where whole-genome SNP assays with adequate genomic resolution for association mapping due to rapid LD decay are still a long way from being available. In such cases well-conceived 'omics' studies may act as a magnet to help find the needles in the haystack that is the genome.

The use of microarrays is now a firmly established method, with transcriptomics being the most mature of the now multitude 'omics' fields. Expression microarrays allow the parallel profiling of most or all genes in a genome, and sensibly designed biological experiments allow the application of microarrays to identify patterns of gene expression associated with a trait of interest. Such traits can be temporal, spatial, or adaptive. As well as conducting individual experiments aimed at answering specific hypotheses, the concentration on MIAME (Minimum Information About a Microarray Experiment, [26]) compliance has enabled the use of meta-analysis approaches that aim to identify patterns across many experiments, or across species. Much effort has been put into the development and subsequent refinement of methods to isolate biologically meaningful information from the background noise [27-30].

An interest in the application of clustering methods to microarray data, whereby groups of genes exhibiting similar expression profiles are identified, was borne from the regulon concept whereby it is expected that a single 'master regulator' (typically a transcription factor) will control the expression of many genes as an efficient way of initiating gene expression 'programs'. For example, leaf development can be considered as a progression through multiple, successive, modules of a developmental program running from primordia initiation to leaf maturity. We have previously shown that some such successive program modules can be identified by profiling gene expression of a field-grown aspen throughout the growing season in multiple years. This identified modules that could broadly be defined as 'cell division', 'cell elongation', and 'differentiation/maturation' [31]. Identifying such clusters (regulons/transcriptional modules, hereafter referred to as transcriptional modules) also allows inference about the function of unknown genes [32]. Clustering was first carried out at the level of an individual experiment using mainly hierarchical clustering [33], k-means clustering [34] and Self Organising Maps (SOMs, [35]) with other methods following. Interest has now extended to identifying transcriptional modules across more extensive collections of expression data [36-41] as well as to the application of network theory and analysis to reconstruct gene regulatory networks [42-47].

We have previously established UPSC-BASE, a database repository with integrated transcriptomics analysis tools [48] for our own *Populus *cDNA microarray platform, which now contains by-far the largest collection of transcriptomics data available for *Populus*. Here we show how this data can be mined using a meta-analysis and cross-validation approach (using external datasets and cross-experiment comparisons) to identify candidate genes involved in leaf development. Our aim was to answer the questions a) by comparing the expression profiles of contrasting developing tissue types, can we identify genes that are of particular importance in leaves? b) Using the available microarray data, can we identify transcription factors and 'leaf transcriptional modules'? c) Can we use the results to identify meaningful patterns associated with the process of leaf development? This work represents the first meta-analysis of *Populus *transcriptomics data and is one of only a small number of cross-experiment transcriptomics studies in plants.

## Results

### Identifying genes of importance to leaves using the UPSC-TC experiment

**Figure 1:**
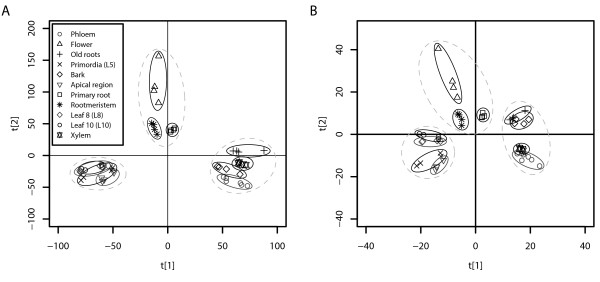
**Principal component analysis overview of gene expression in different tissues of Populus tremula × tremuloides 'T89**. PCA overview of the expression of the 14446 genes (A) and of the only the 955 transcription factors (B) represented on the POP2 microarray in different tissue types of *Populus tremula *× *tremuloides *'T89'.

We were first interested to see whether different tissues types could be distinguished based on their expression profiles and if so, whether a set of leaf-specific genes could be identified for further down-stream characterisation. The UPSC-TC (Tissue Comparison) experiment profiled gene expression in leaves (of various ages), wood tissues (phloem, xylem), root tissues, flowers and the three meristems (shoot apical, root, cambial) of hybrid aspen (*P. tremula *× *P. tremuloides *'T89'), with each tissue type being profiled against a common reference formed by pooling equal quantities of RNA from all tissues. We used Principal Component Analysis (PCA) to provide a visual overview of the UPSC-TC dataset. PCA is described as an unsupervised analysis method because no *a priori *information regarding sample classes (here, tissue type) is given and any sample grouping seen is therefore an inherent feature of the data. Figure 1 shows that tissue types could be differentiated from each other on the basis of gene expression. Figure 1A shows the results for all gene models represented on the POP2 microarray (14446 gene models) with Figure 1B showing all transcription factor gene models (955 gene models). Both figures show the same clear separation of tissue types. Here we concentrate further only on the leaf group, results for the other tissues will be described elsewhere.

**Figure 2:**
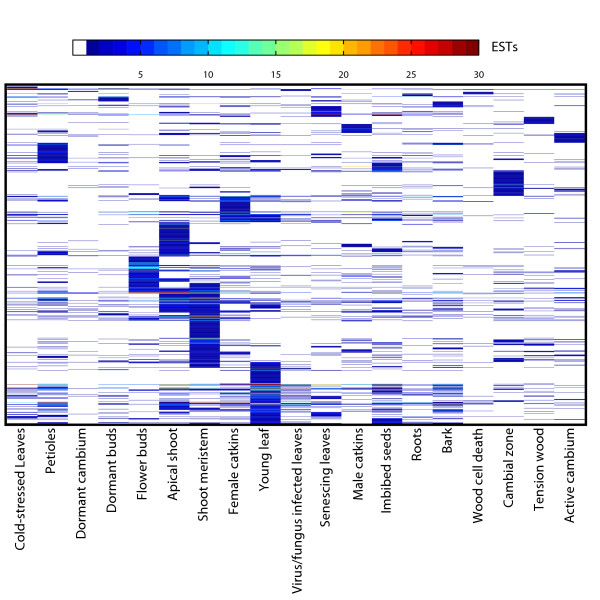
**Digital northern heat map representation of leaf genes in PopulusDB EST libraries**. Heat map representation of a digital northern to examine the library distribution of the identified leaf gene set. The results are based on the data contained in PopulusDB [53] and genes are arranged on the basis of cluster analysis to aid visual interpretation.

**Figure 3:**
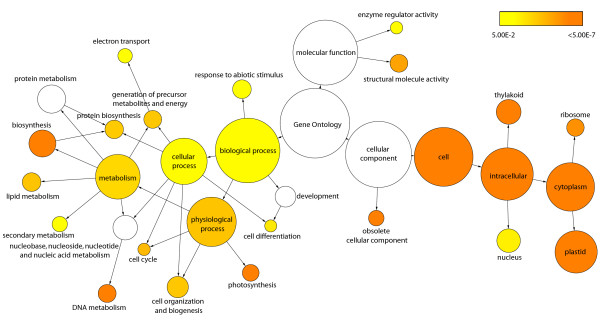
**Gene ontology category over-representation in leaf genes**. Statistically over-represented Gene ontology categories within the leaf gene set. Statistical significance was calculated using an hypergeometric test with an FDR-adjusted cut off of 0.05. The size of each circle indicates the number of genes within a category and circles are shaded based on significance. Categories in white were non-significant.

Having shown that tissue types could be meaningfully distinguished from each other based on expression data, we then wanted to identify those genes that were most representative of leaves. Orthogonal Projection to Latent Squares (OPLS) is a supervised multivariate linear regression method [49] and is a supervised method in that it makes use of *a priori *information to guide the analysis [50], e.g. tissue classes, as in the present case. The unique property of OPLS as a regression method lies in its innate ability to separately describe information in the data table that is related to the modelling aim (e.g. to discriminate between different classes) and other systematic trends (e.g. instrumentation drift or batch variability etc.). OPLS analysis identified 1116 genes that were markers useful in the discrimination of leaf from non leaf tissues when considering all gene models represented on the POP2 array (Additional file 1). Having identified these genes, we wished to examine their biological function. Figure 2 shows a heat map visualisation of a digital Northern – based on EST frequencies in different libraries (Sterky et al. 2004) – of the OPLS-generated leaf gene list and shows that the greatest prevalence of genes was found in the shoot meristem, young leaf and apical shoot libraries. There is also distinct under-representation within the dormant cambium and wood cell death libraries. The active cambium library is not over-represented with genes, which suggests that the genes we have identified are not simply general markers of high cell division. A distinct band of genes can be seen in the flower bud library but this is perhaps not surprising considering that flowers are modified leaves originating from the same apical meristem. To provide information on the functional role of the identified leaf genes we tested for GO (Gene Ontology [51]) categories that were significantly over-represented. This analysis showed enrichment for both Biological Processes and Cellular Components associated with photosynthesis (Figure 3). There was also enrichment of categories that are more likely involved in developmental processes such as the Biological Process categories Cell Differentiation, Cell Organization and Biogenesis, and Cell Cycle. We repeated the GO analysis after removing genes in categories associated specifically with photosynthesis and chloroplasts and this revealed even greater enrichment of categories associated with developmental events, including the Biological Process category of Leaf Development (data not shown).

As transcription factors represent master controls within developmental programmes and are likely targets for explaining phenotypic diversity, we were particularly interested to see which transcription factors were over-represented when considering the frequency of incidence of ESTs representing gene models for the leaf libraries of PopulusDB and which transcription factors were important in separating leaf from non-leaf tissues in the UPSC-TC dataset. To this end, we used the PopGenIE [52] CatFisher tool to identify transcription factor gene models that were over-represented in the young leaf, apical shoot and shoot meristem libraries of PopulusDB [53] (Table 1a). The Fisher's exact test (the statistical test underlying the CatFisher tool) identified three transcription factors over-represented in leaf EST libraries (a CCAAT-HAP3 in the apical shoot library, and a CCAT-HAP5 and C2C2-YABBY in the shoot meristem library). An additional test to identify transcription factor families (rather than individual transcription factors) that were over-represented in leaf libraries showed that the C2C2-YABBY and CCAAT-HAP5 families were over-represented in the shoot meristem library and the C2C2-CO-like family in the young leaf library. An OPLS analysis considering only the transcription factors represented on the POP2 arrays (955 genes) identified 100 transcription factor gene models as being useful for distinguishing leaves from non-leaf tissues (Additional file 2).

**Table 1:** Transcription factors preferentially expressed in leaves

a) Digital Northern								
**EST library**	**Family**	**Gene Model**	**Protein ID**	**Linkage group**	**Start bp**	**End bp**	**Clostest ATG**	**Annotation**
K	CCAAT-HAP3	estExt_fgenesh4_kg.C_LG_IX0001	814069	IX	422283	424607	At2g27470	CCAAT-box binding transcription factor subunit HAP3-related
T	CCAAT-HAP5	eugene3.00130826	571462	XIII	7433295	7434557	At1g51060	Histone H2A
T	C2C2-YABBY	grail3.0018017701	646464	III	10874457	10877479	At2g45190	AFO/YAB1
b) Microarray								
	bHLH	gw1.29.118.1	423606	scaffold 29	2272702	2274494	At5g46690	loop-helix (bHLH) family protein
	C2C2-YABBY	gw1.XVI.2137.1	256198	XVI	4660471	4663832	At2g26580	YAB5
	C2C2-YABBY	gw1.I.9758.1	181158	I	11002077	11007440	At2g26580	YAB5
	C2C2-YABBY	grail3.0018017701	646464	III	10874457	10877479	At2g45190	AFO/YAB1
	C2C2-YABBY	grail3.0035001101	663774	XIV	1201250	1204103	At2g45190	AFO/YAB1
	C2C2-YABBY	estExt_Genewise1_v1.C_1270153	744044	scaffold 127	432420	435374	At2g45190	AFO/YAB1
	CCAAT-HAP5	gw1.133.51.1	268609	scaffold 133	194018	194708	At5g27670	Histone H2A
	GRF	eugene3.00021070	551755	II	8750087	8753351	At4g37740	GRF2
	HB	estExt_fgenesh4_pm.C_LG_II1004	830518	II	19705723	19710899	At1g05230	homeobox-leucine zipper family protein
	HMG	gw1.I.8656.1	180056	I	6841319	6843651	At4g11080	High mobility group (HMG1/2) family protein
	HMG	estExt_fgenesh4_pg.C_LG_VII1313	820147	VII	12719268	12721109	At5g23420	High mobility group (HMG6)
	MYB	gw1.147.131.1	271563	scaffold 147	383655	384226	At1g22640	myb family transcription factor (MYB3)
	MYB	estExt_fgenesh4_pm.C_LG_VI0283	831892	VI	5731437	5734378	At2g37630	AS1
	MYB	gw1.XII.82.1	421622	XII	9811004	9812387	At5g14750	Werewolf1 (WER1)
	ZF-HD	gw1.IV.4567.1	199478	IV	11561033	11561668	At2g02540	zinc finger homeobox family protein (ZFHD4)
	ZF-HD	fgenesh4_pg.C_LG_V001422	761370	V	15831484	15832356	At2g18350	zinc finger homeobox family protein ATHB24)
	ZF-HD	gw1.41.334.1	287849	scaffold 41	1309979	1310581	At4g24660	zinc finger hmeobox family protein ATHB22/MEE68)
	ZF-HD	gw1.V.4670.1	209269	V	8372094	8372846	At5g65410	zinc finger homeobox family protein (ZFHD2)

Transcription factors significantly over-represented in leaf EST libraries contained in PopulusDB [53] and identified as being informative in the discrimination of leaves from non-leaf tissues in the UPSC-TC experiments using Orthogonal Projection to Latent Sqaures.

As well as transcription factors being important regulators, it has also become clear that miRNAs play a key regulatory role, especially in restricting the zone of expression of genes thought to be involved in development. Using the target site predictions provided in [54], we tested the OPLS leaf genes to see if any of the miRNAs currently in miRBase [55] were over-represented for predicted target sites within this gene list. As not every gene model is represented on the microarrays used, we first restricted the target site prediction dataset to only those gene models profiled. We additionally had to translate the pre v1.0 gene models used in [54] to their v1.1 equivalents. Five of the seven members (c-g) of the ptc-MIR396 family were found to be over-represented for predicted target sites within the OPLS leaf genes, with no other miRNAs showing over-representation. Of these five significant family members, ptc-MIR396g was by-far the most significant with seven of its 11 predicted targets being found within the OPLS leaf gene list. Examination of the genomic location of these miRNAs in the PopGenIE Browser revealed that ptc-MIR396c,f,g are located within clusters of QTL for leaf development traits on LG III, VI and VII respectively. The other members of MIR396 do not collocate to leaf development QTL and additionally, none of the predicted target genes collocate with leaf QTL. As another route to examining the potential role of miRNAs in leaf development, we examined the number of predicted miRNAs targetting the OPLS leaf genes (Additional file 3). Six genes were predicted to be targetted by seven miRNAs, with five of those genes having maximum homology to *Arabidopsis Growth Regulating Factor *(*GRF*) genes, which are discussed below. For the five genes annotated as *GRFs*, the seven miRNAs predicted to target them are the seven members of the ptc-MIR396 family. The sixth gene is predicted to be targetted by seven members of ptc-MIR169 (i-m, o,p). This gene is of unknown function and has no reported *Arabidopsis *mutant phenotypes in TAIR currently. We additionally performed a broader examination of all genes (not just the leaf genes or those represented on the UPSC cDNA microarray) predicted to be highly targetted by miRNAs to see which GO Biological Process categories were over-represented. For this analysis we used only genes predicted to be targetted by more than 10 miRNAs using the entire set of predicted miRNAs from [54] as well as only the 'official' miRBase miRNAs. In both cases, there was an almost exclusive and dramatic over-representation of gene involved in developmental processes and pattern formation (Additional file 4 shows the results of the analysis using the ptc-miRBase miRNA subset).

### Identifying and characterising transcriptional modules in leaves

**Figure 4:**
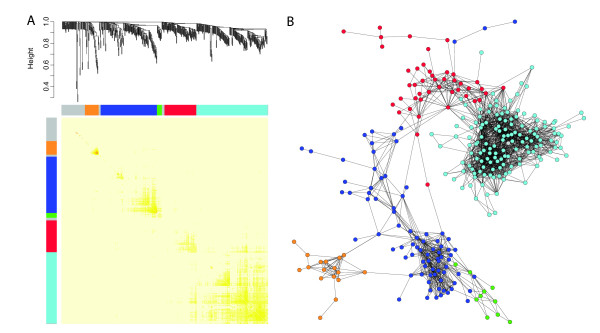
**Transcriptional module identification and network visualisation in the UPSC-Leaf microarrays**. A Average linkage hierarchical clustering dendrogram of the UPSC-Leaf dataset. Five transcriptional modules were detected. Modules were assigned colours as indicated by the bar below the dendrogram. Rows and columns in the heat map represent genes in a symmetric fashion. Colour intensity represents connection strength between two genes from red (the strongest connection) to pale yellow (no connection). B Network visualisation based on pair-wise Pearson correlations. Nodes represent genes and are connected by edges where significant correlation between genes exists. Nodes are coloured based on the five modules detected in A. Edges are drawn for correlations > 0.7. Unconnected nodes are not plotted and nodes with only individual pair-wise connections that were not connected to the central network were removed.

Having identified a set of leaf marker candidate genes, we were interested to see if transcriptional modules could be indentified within this set of genes as a means of dissecting regulatory control. Transcriptional modules were identified in the OPLS leaf gene set using the UPSC-Leaf dataset using TOM (Topological Overlap Matrix) weighted-co-expression analysis [39]. The UPSC-Leaf dataset is a collection of 642 microarrays from 22 experiments profiling leaves in a range of developmental stages, biotic and abiotic stress treatments, and transgenic lines (see Methods for details), the results of which are visualised in Figure 4A. Figure 4A shows the resulting hierarchical co-expression tree generated by the analysis. Each major branch of the tree is defined as a transcriptional module and a colour is then assigned to the genes within those modules (Additional file 5). Another common way of analysing and representing such data is to perform a pair-wise Pearson correlations analysis (where the correlation between all gene pairs is calculated) and to then plot these results as a network diagram. We show such an analysis in Figure 4B, in which we have coloured the genes based on the module they were assigned to using the TOM analysis presented in Figure 4A. It is clear that the network visualisation of the correlation structure reveals the same grouping of genes as the TOM analysis. The network view also shows that some genes are clearly more connected than other genes and these are likely to be key regulatory controls within the transcriptional modules. The most highly connected gene within each module of the TOM analysis is listed in Table 2. In Figure 4A, the grey genes are those that were not members of a module and GO over-enrichment analysis of this group of genes did not find any significantly over-represented categories (a negative control). We tested each module for GO category over-representation in order to ascribe a broad functional role to each module. Based on these results, the turquoise module could be described as 'chloroplast/photosystem', blue as 'DNA replication and structure', red as 'intracellular/organelle', green as 'protein biosynthesis' and orange as 'secondary metabolism'.

**Table 2:** The most highly connected gene within transcription modules

**Module**	**Gene model**	**Protein ID**	**Linkage Group**	**Start bp**	**End bp**	**Closest ATG**	**Annotation**
green	estExt_fgenesh4_kg.C_LG_X0057	814174	X	16689588		At5g46020	similar to cupin family protein
blue	estExt_fgenesh4_pm.C_LG_V0721	831725	V	17784177		At1g76540	cyclin dependent kinase B2:1
red	estExt_fgenesh4_pg.C_LG_XII1021	823432	XII	11985750		At4g25050	acyl carrier protein 4 (ACP4)
turquoise	estExt_fgenesh4_pg.C_440087	826955	scaffold 44	1069077		At1g55150	DEAD box RNA helicase (RH20)
orange	estExt_fgenesh4_pg.C_1580005	828416	scaffold 158	67532		At5g07990	Transparent Testa 7 (TT7), required for flavonoid 3' hydroxylase activity
grey	estExt_fgenesh4_pg.C_LG_X1117	822127	X	12350943		At1g09740	ethylene-responsive protein, similar to Universal Stress Protein (USP)

The most highly connected gene within each transcription modules identified using average linkage hierarchical clustering. Module colours refer to Figure 4.

### Cross-experiment and cross-species validation

**Figure 5:**
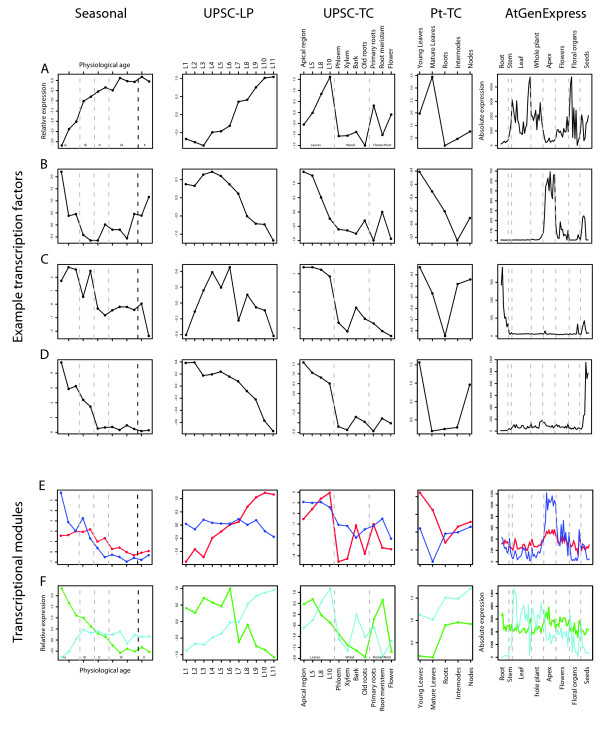
**Cross-experiment validation of leaf development candidate transcription factors and modules**. A-D Expression of selected transcription factors with developmental expression patterns in four *Populus *microarray experiments (Seasonal data of [31], UPSC-TC experiment, UPSC-LP experiment, Pt-TC experiment) and of the best BLAST hit ortholog in the *Arabidopsis *AtGenExpress developmental baseline experiment [56]. The genes are gw1.VII.2982.1, estExt_Genewise1_v1.C_290455, gw1.XII.82.1, gw1.41.334.1 respectively. E-F Expression of the most highly-connected gene for transcriptional modules with a developmental expression trend identified by Topological Overlap Matrix weighted co-expression analysis. Genes are coloured based on their module colour in Figure 4. Blue, estExt_fgenesh4_pm.C_LG_V0721; Red, estExt_fgenesh4_pg.C_LG_XII1021; Green, estExt_fgenesh4_kg.C_LG_X0057; Turquoise, estExt_fgenesh4_pg.C_440087.

Having shown that we could define candidate transcription factors and transcriptional modules within leaves, we then wanted to see a) how consistent their expression was across different experiments profiling leaf development, and b) which of the identified genes had patterns of expression suggesting a role in leaf development. As a means of representing the expression profile for each transcriptional module, the expression of the most highly connected gene and of the gene most closely correlated to the Eigen gene (the first principal component of the module) of each identified module was plotted in the seasonal dataset presented in [31](UMA-0032), the UPSC-TC, UPSC-LP (Leaf Primordia), and Pt-TC (*Populus trichocarpa *Tissue Comparison) experiments (see Methods for details), as well as of the *Arabidopsis *orthologs (defined by best BLAST hit results reported in [53]) in the AtGenExpress developmental baseline dataset [56]. All transcription factors identified using OPLS were similarly plotted in the same datasets. We then visually screened through these plots to identify those genes/modules with an expression pattern suggesting they may be involved during stages of leaf development or that could serve as markers of leaf maturation. Manually identified subsets of the most interesting of these are shown in Figure 5 with parts A-D representing transcription factors and parts E-F the most highly-connected gene of each transcriptional module. The most highly-connected and most closely correlated gene to the Eigen gene for each module showed very similar expression patterns for all modules and we have therefore only plotted the most highly connected genes. Screening of the OPLS list of transcription factors identified 18 (Table 1b) as having particularly interesting expression patterns during leaf development and these are detailed in Table 1. Five of the transcription factors are from the C2C2-YABBY family, four from the ZF-HD family, and three from the MYB family. As there are a number of examples of *Arabidopsis *orthologs being represented by multiple *Populus *gene models in Table 1, it seemed likely that these could potentially represent *Populus *paralogs. The data presented in Figure 2 of [57] concerning the genome duplication event in *Populus *does not suggest that these gene models are likely to be paralogous copies resulting from the duplication event and this assumption is supported by the Plant Genome Duplication Database [58] conserved syntenic block information displayed in the PopGenIE Browser.

In Figure 5A–D it can be seen that some of the selected transcription factors have expression patterns that were highly consistent between *Populus *and *Arabidopsis *(A, B), while other examples (C, D) showed contrasting expression profiles. This is possibly due to differentiation of function between the two orthologs but is also possibly due to the fact that the ortholog identified by best-BLAST score analysis is not the true ortholog. Here we do not attempt to differentiate between the two possibilities, we simply define orthologs based on our analysis pipeline. Figure 5A shows an example of a gene that is a good marker of leaf maturity, with low expression at early leaf development stages in all *Populus *experiments and with high expression particularly in older leaves and floral organs in *Arabidopsis *and low expression in the apex samples. Figure 5B shows the opposite case – a gene that is a good marker for early leaf development stages, dropping rapidly in expression as leaves mature and with a distinct peak of expression in the *Arabidopsis *apex samples and no expression in older leaf samples. Figure 5C–D shows two additional markers of early leaf development stages in *Populus *but in both cases, expression in the *Arabidopsis *apex samples is either absent (C) or very low (D). In the case of Figure 5C, a distinct and almost unique peak is seen in *Arabidopsis *roots, which contrasts to the *Populus *expression pattern of this gene. In Figure 5D, a distinct peak is seen in *Arabidopsis *seed samples but not in the apex or leaves, which contrasts to the expression pattern observed across the *Populus *experiments, where expression is only high in young leaves and in internode samples of the Pt-TC experiment (which contain a secondary vegetative meristem). *Populus trichocarpa *shows weak apical dominance and so it is unsurprising that expression of a gene that is high in young leaves and in the shoot apex would also be high in internode samples. In general, we found that ~60% of genes had broadly similar expression patterns between *Arabidopsis *and *Populus *with ~30% showing more than broad similarity. The remaining 40% of genes showed a range of divergence between *Populus *and *Arabidopsis*, with some (such as those in Figure 5C–D) showing distinctly contrasting patterns between to the two species.

As an additional means of examining how robust the results from the UPSC-TC experiment were, we performed an OPLS analysis on the Pt-TC experiment, which compares different tissue type of *Populus trichocarpa *using a different array technology, to examine the degree of agreement between the two datasets. A direct comparison of the results from the two microarray platforms is problematic due to the considerably greater number of gene models represented on the Nimblegen microarray used for the Pt-TC experiment (54,794 compared to 15,789). We therefore performed the OPLS analysis for this experiment on the entire set of 54,794 nuclear gene models and then restricted the gene list obtained to only those gene models represented on the UPSC POP2 array (we consider this to be a more stringent approach than limiting the dataset to only those genes present on the UPSC POP2 array before performing the OPLS analysis). The union between the restricted Pt-TC and the UPSC-TC gene lists was then examined. 452 gene models (40.5%) were identified in common (Additional file 6).

### An example extended-application of the results

**Figure 6:**
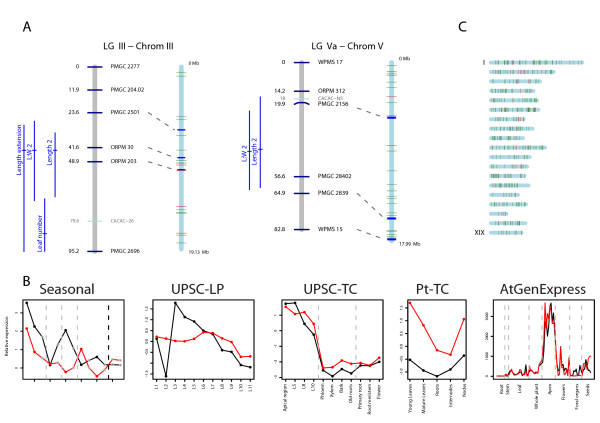
**Candidate gene collocation to QTL for leaf development**. Example extended application of identified leaf candidate genes. A – Collocation of genes that were highly helpful for the discrimination of leaf tissue from other tissue types to QTL for leaf length in the control condition from the results of [9]. Linkage groups are shown as grey bars and chromosomes as blue bars. SSR markers are indicated by black text and AFLP markers by grey text. Dashed grey lines connect the location of SSR markers in the linkage group and chromosome. QTL are plotted to the left of the linkage group with a horizontal line showing the peak F score location and confidence intervals plotted as verticals lines. The location of genes is indicated by horizontal lines on the chromosome with transcription factors represented by red lines and other genes by green lines. B – Expression of the two transcription factors located between flanking markers from A in four *Populus *microarray experiments (Seasonal data of [31], UPSC-TC experiment, UPSC-LP experiment, Pt-TC experiment) and of the best BLAST hit ortholog in the *Arabidopsis *AtGenExpress developmental baseline experiment [56]. The transcription factor from LG III (grail3.0018017701, AT2G45190) is plotted in black and that from LG V (gw1.V.4670.1, AT5G65410) in red. C – Chromosomal location of genes (green vertical lines) and transcription factors (red vertical lines) that were informative in the discrimination of leaves from other tissues identified using Orthogonal Projection to Latent Sqaures.

We were keen to show how the results presented here can be used in a genetical genomics context. Using the QTL data from [9] as an example, we plotted the collocation of QTLs to genes and transcription factors identified using OPLS from the UPSC-TC experiment (map data to allow plotting was supplied by Tuskan J, pers. comm.). We decided to only consider QTL mapped in the control condition of [9], and this resulted in seven LGs with QTL for leaf development traits (area expansion, length, width and extension for both, and mature leaf area). By considering the underlying gene(s) for a QTL to exist between the closest flanking sequence-based markers (the only link we have between the genetic and physical maps), we identified and plotted the expression of all collocating candidate genes from the current study within those seven QTL regions across the five experiments presented in Figure 5. We then visually screened this set of graphs to identify candidates with an expression profile suggesting a role during leaf development (so high expression at young leaf ages, dropping rapidly as leaves mature). This resulted in two particularly good candidates, which both collocated to QTL that appear to be specific to leaf length (Figure 6A). The expression profile of these two candidates, which are both transcription factors (Figure 6B), is indicative of a gene involved in early stages of leaf development (principally during the cell division phase), and both genes have highly-similar expression profiles across all five experiments considered, including in *Arabidopsis *(Figure 6B, end graph). Both of these genes were also identified in the transcription factor analysis described above. By additionally viewing these genes in the PopGenIE Browser, we could also see that these regions of the genome contain QTL for leaf development associated traits in a number of QTL studies performed on this population. The gene on *Populus *LG III is the ortholog of the *Arabidopsis Abnormal Floral Organs*, a YABBY transcription factor (*YAB1*) known to be involved in abaxial cell fate specification [59-61], the protein of which is located in the leaf abaxial epidermis. The gene on LG V is a zinc finger homeobox family protein of unknown function. AT5G65410 collocates to a QTL for a number of leaf dimension traits on chromosome five in *Arabidopsis *(LQTL-3, [10]) if a 1:1 relationship between genetic and physical map distances is assumed. Using the same approach, we also found that three members of the miRNA family ptc-MIR396, all members of which were found to have over-representation of target sites within the OPLS leaf genes, collocate to clusters of QTL for leaf development traits in both [9] and the additional experiments represented in the PopGenIE Browser. This leads to the interesting possibility that a miRNA, or multiple members of a family, could be the causative loci of a QTL.

To examine the distribution of candidate genes across the genome, we plotted their locations to allow a visual overview (Figure 6C). There appear to be two potential clusters of genes on LG VI and another, smaller, cluster on LG XIV. Perhaps interestingly (but certainly a coincidence of note), *AS1 *is located at the site of the first cluster on LG VI, *ER *at the site of the second and *AN *within the cluster on LG XIV. The above example does not provide any evidence that the genes we have selected in any way have a causal role in controlling leaf development, size or shape. It does, however, provide a useful and interesting example of how the dataset we present can be used and integrated within a genetical genomics context.

## Discussion

After an early wave of enthusiasm for high-throughput transcriptomics, attention is shifting somewhat towards the next steps up the ladder of the central dogma, proteomics and metabolomics, and to the integration of information across all levels i.e. the systems biology approach. This shift in attention is partly due to the fact that control at the level of the transcriptome tells only part of the whole story in the plot of genotype to phenotype. However, transcriptional data is still important, telling which genes are being transcribed even if they are eliminated as characters from the plot at later stages by factors such as miRNA degradation. The criticism now commonly placed on transcriptomics (i.e. the potential for transcription not to represent protein activity) will, of course, hold equally true when proteomics data is examined in increasing detail; knowing which proteins are present tells you nothing about their half life, rate of replacement or side-branch decoration.

Here we have used an extensive set of transcriptomics data from a diverse range of sources, covering different microarray platforms, species of *Populus *as well as *Arabidopsis *to identify genes that are actively transcribed during the process of leaf development. A similar starting point to ours was previously reported for *Arabidopsis*, where it was found that different tissue types could be differentiated on the basis of gene expression data [56]. However, only four leaf-specific markers were identified. We believe that *Populus *represents a more suitable model for answering these questions as the degree of tissue differentiation is greater than in *Arabidopsis *(contrast the trunk of a *Populus *tree to the stem of *Arabidopsis*), a fact that should increase the resolution with which tissue-specific expression patterns can be discriminated. Here we were also able to clearly separate tissue types on the basis of gene expression but with greater within tissue-type resolution than was possible using *Arabidopsis*. Figure 1 shows that leaf samples form a unique group that is distinct from other tissue types. A second group was found for 'wood' samples, including old roots (which were most similar to bark), and a third, less distinct, group containing young roots and flowers. When considering only those gene models predicted to have transcription factor activity, we could see further differentiation within the three main groups (Figure 1B). EST library representation (Figure 2) and functional enrichment analysis (Figure 3) of gene models that were highly representative of leaves in comparison to other tissues identified apical shoot and young leaf EST libraries and GO categories for photosynthesis and chloroplasts (as one would expect) but also a number of categories likely to be involved in leaf development. As functional enrichment was tested for in a list of genes that are more representative of leaves than other tissues, these genes are not simply general markers of high cell division/expansion but are more specifically involved in those processes primarily in leaves. We were interested primarily not in identifying genes that are highly representative of leaves *per se *but more specifically, genes that are involved in leaf development or that could serve as developmental markers. Our own interest in this approach is in the identification of candidate genes for association mapping in the SwAsp collection of Swedish aspens [62]. We also wanted to develop a set of developmental marker genes as we have previously shown that the magnitude of transcriptional regulation during development is greater than that induced by changes in the weather for a field-grown aspen [31] and that the induced transcriptional response to a treatment can be masked by developmental alterations induced by the treatment [63]. The availability of developmental markers would allow rapid comparisons to be made between leaves thought to be at the same developmental state to confirm whether like is being compared with like. As many more genes than can be sensibly used for downstream applications were identified in the OPLS method, we took the approach of concentrating on transcription factors and of identifying transcriptional modules within our list of candidate genes as a means of selecting a subset of genes that would maximise the information content. We were able to identify transcriptional modules (Figure 4A) that appear to be robust (Figure 4B) and for which broad biological function could be assigned to. For each module, we plotted the expression of the most highly connected gene across a range of microarray data sets in both *Populus *and *Arabidopsis *(Figure 5E–F). As can be seen, the green and blue modules appear to be important in early stages of leaf development, the red module in later stages (differentiation rather than cell production), and the turquoise module important in later development and in mature leaf functioning. These patterns of expression make sense when considered alongside the functional classification for each module: The green (protein biosynthesis) and blue (DNA replication and structure) are highly expressed in young leaves that are involved primarily in cell division, the red (organelle/intracellular) module then having a later peak as cells begin to expand and differentiate, with the latest peak in the turquoise (photosynthesis/chloroplast) module, which peaks and remains high as differentiation nears completion and expansion reaches an end, marking the point where leaves switch from sinks to sources. This progression through development is also revealed by the connectivity seen between modules in Figure 4B, with there being a progression in developmental time from the bottom (green and blue) to the top (turquoise) of the diagram. The orange (secondary metabolism) module has a more variable expression profile (data not shown) and is also the least inter-connected of the network modules (Figure 4). Although a regulatory role cannot easily be ascribed to each of these genes (Table 2 – one would expect the most highly connected gene to be the best candidate for being the module regulator gene), for some a regulatory role does seem plausible. For example, the most highly connected gene in the blue module is estExt fgenesh4 pm.C LG V0721, the ortholog of AT1G76540, a cyclin-dependant kinase involved in regulation of the G2/M transition of the mitotic cell cycle [64]. Most of the genes identified in Table 2 do not have reported under- or over-expression phenotypes and are currently of undetermined function. There is currently little information on how transferable and conserved such complex developmental transcriptional modules identified from transcriptional data are likely to be, especially in plants.

Due to their key role in controlling developmental programs and their ability to influence the expression of a number of downstream targets (*trans *effects), we concentrated our analysis on transcription factors. Transcription factors of interest were selected using OPLS and EST library over-representation tests. This combined approach provides evidence that the C2C2-YABBY, CCAAT-HAP3 and 5, ZF-HD, and MYB families of transcription factor families are of particular importance in leaves, with C2C2-YABBY being the most highly represented family. YABBY transcription factors are known to be involved in the process of abaxialisation [59-61,65-67] and ectopic expression of YABBY genes is sufficient to cause the abaxialisation of adaxial epidermal tissues [59-61]. Five gene models in the C2C2-YABBY family were identified by the OPLS analysis of the UPSC-TC experiment with one of those (grail3.001817701) also identified by the EST library over-representation test. Three of the five gene models are orthologs of AT2G45190 (*AFO*/*FIL*/*YAB1*) and the other two of AT2G26580 (*YAB5*). Although no functional evidence is available for these genes in *Populus *yet, homologous *Arabidopsis *genes have been chacterized. [61] report that there was no observable leaf phenotype in the *fil *single mutant but suggest this is the result of redundant activities of other members of the YABBY family. [60] report aberrant leaf phenotypes for 35S over-expression lines of *FIL*, with strong over-expression apparently being lethal and weaker over-expressing lines showing the formation of wrinkled leaves. The expression of *YAB5 *was recently suggested to be negatively modulated in the adaxial domain by *AS1 *and *AS2*, two genes critical for the development of properly expanded leaves [68]. Two of the identified transcription factors have mutant phenotypes reported in TAIR. Eugene3.00021070 is the ortholog of AT4G37740, *Growth-Regulating Factor 2*, the mutants of which have smaller leaves [69], and which is one of the predicted targets of the ptc-MIR396 miRNA family that we found to be over-represented for target sites in the OPLS leaf genes. EstExt fgenesh4 pm.C LG VI0283 is the ortholog of *ASYMETRIC LEAVES 1 *(*AS1*, AT2G37630), which is homologous to the Antirrhinum *PHANTASTICA *(*PHAN*) and maize *ROUGH SHEATH2 *(*RS2*) genes [70-72] and which acts as a negative repressor of class I KNOTTED1-like homeobox (*KNOX*, in particular *KNAT1 *and *KNAT2*) genes and that shows genetic interaction with *SHOOT MERISTEMLESS *(*STM*; [70]) and *BREVIPEDICELLUS *(*BP*; [18]). *as1 *mutants have highly lobed leaves, occasionally forming ectopic shoots on leaves [70,73] show that *AS1 *and *AS2 *together with *ERECTA *(*ER*) are involved in the establishment of adaxial-abaxial polarity (with *ER *appearing to protect the *AS1*/*AS2 *pathway from heat stress; [74]). [75] show interaction of *AS1 *with *CUP-SHAPED COTYLEDON *(*CUC*) 1, a gene involved in shoot apical meristem formation. It would therefore appear that *AS1 *is a key and central player in leaf formation and pattern development. Through a combination of the various analyses undertaken in this study, it would appear that miRNA family ptc-MIR396 and the *GRF *gene family may represent interesting targets for follow-up studies.

As a means of indicating how generally-applicable and relevant our finding are, we were interested to see what degree of overlap would be found between the UPSC-TC and Pt-TC experiments if the same OPLS analysis was carried out on the Pt-TC experiment. We did not expect a substantial overlap as the two experiments have some key differences: The UPSC-TC experiment samples tissues to a greater resolution and, for the leaf samples, includes a set of leaves that are considerably younger than the young leaf samples in the Pt-TC experiment (see GEO for sample details of the Pt-TC experiment). However, 452 (40.5%) of genes were found in common between the UPSC-TC and Pt-TC experiments, which suggests that the analysis method is robust and applicable beyond the samples contained in the original experiment (data not shown). This also suggests that our identified genes are likely to be functionally important within the SwAsp aspen trees that we wish to use for future association mapping, which was a major motivation for this undertaking rather than simply selecting candidate genes on the basis of published forward genetics screens.

As an example of the potential uses of the information presented by this work, we examined the collocation of identified candidate genes and transcription factors to QTL for leaf development that we had mapped in previous work [9] and to those additional QTL available in the PopGenIE Browser. This allowed us to identify two collocating transcription factors, one of which (*AFO*) has functional support for a role in leaf development in *Arabidopsis *[59-61]. We have also shown that multiple members of a miRNA family identified as targeting a number of genes within the OPLS leaf gene set additionally collocate to QTL for leaf development, raising the potential for miRNAs being the underlying genetic loci of a QTL.

There are at least six publications containing QTL data for leaf related traits in the mapping population considered here alone (and a greater number if one considers all results published in any *Populus *mapping population), but in no case (our own publication included) would this collocation analysis have been possible using only the published data. We would therefore strongly suggest that a MIAME-equivalent standard should be established for the publication of QTL results to ensure that map, QTL, and phenotype data are always made publicly available as a requirement for the publication of QTL results, especially with the increasingly availability of eQTL results in addition to more classical phenotypic QTL data as this will ensure that such cross-species, comparative genomics studies are achievable in the same way that they are in the transcriptomics field thanks to standards compliance.

## Conclusion

We have shown that a large, diverse collection of microarray data can be used in a combined-analysis approach to identify candidate genes involved in the processes of leaf development. Network-clustering analysis on a set of genes that are highly-representative of leaves identified modules with distinct patterns of expression associated with leaf development and functional enrichment analysis of genes within each module revealed that the pattern of expression between the modules makes biological sense in the context of their likely functional roles. Our cross-experiment and cross-species validation approach showed that identified genes, particularly the transcription factors, have patterns of expression that suggest they represent a robust set of candidate genes involved in leaf development. The analysis approach used identified genes with proven functional roles in leaf development in *Arabidopsis *and other species, suggesting that our method is biologically robust and the results meaningful. This suggests that the identified genes of as-yet unknown function are sensible targets for further targeted functional characterisation or as candidates for association mapping, as well as providing a set of genes that can serve as robust markers of leaf developmental age.

## Methods

### UPSC Tissue Comparison microarray experiment

Tissue samples were collected from hybrid aspen (*P. tremula *× *P. tremuloides *'T89') grown under natural light in the greenhouse (UMA-0030) and at eleven positions covering early leaf developmental stages (UMA-0020): the Apex (L1); tissue just below the apex (L2); primordia < 100 μm (L3), 200–250 μm (L4), 500–1000 μm (L5) and 1–3 mm (L6) in diameter; and leaves 1 cm (L7), 2 cm (L8), 3 cm (L9), 4 cm (L10) and 5 cm (L11) long (experiment UMA-0020). In experiment UMA-0030, young root and root meristem samples were collected from hydroponically grown plants. The extended Leaf Primordia series is available in UPSC-BASE as experiment UMA-0020, and we refer to it here as the UPSC-LP experiment.

Material used for the microarrays was prepared and handled as described in [76]. The experiment made use of the POP2 microarrays described in [77] and [53] and for which information can be found in UPSC-BASE [48]. For the UPSC tissue comparison (UPSC-TC) experiment, microarray slides were hybridised against a common reference containing a mixture of all samples. Slides were scanned at 10 μm resolution, using a Scanarray 4000 microarray analysis system scanner (Perkin-Elmer, Boston, MA, USA). Scanner settings were calibrated for PMT (Photo Multiplier Tube) and laser power to ensure even level signal strength for both channels. Spot data were extracted using GenePix version 5.0 (Axon Instruments Inc, Union City, CA, USA). The data output from GenePix was imported into UPSC-BASE, and is publicly available as experiment number UMA-0030. A step-wise normalisation [78] was applied for all slides before further analysis. Based on a Principal Component Analysis (PCA) visualisation of the data (see below) the samples were classified into three groups for OPLS analysis. The three groups were 'leaf' (containing Leaf 8 and 10, Apical region, and Primordia arrays), 'wood' (containing xylem, phloem, bark, and old roots arrays), and 'flower/roots' (containing flower, primary roots, and root meristem arrays).

### UPSC-BASE leaf microarrays

In April 2008, UPSC-BASE contained 22 experiments totalling 642 slides (of which 602 passed our filtering criteria) performed on *Populus spp*. leaves (experiments UMA 0001, 0009, 0011, 0013, 0016, 0020, 0025, 0031, 0032, 0033, 0035, 0036, 0037, 0040, 0042, 0050, 0054, 0063, 0068, 0069, 0078, 0082). These experiments represent a highly diverse set of conditions including biotic and abiotic stress treatments, developmental series and RNAi lines. The data for each experiment were step-wise normalised [78] and filtered to remove spots with an A-value below 8. Finally the separate data files were median aggregated based on gene model and merged to a single matrix. As the microarrays selected were performed on both the POP1 and POP2 platforms, we considered only the set of gene models common to both array platforms. The POP1 array contained 13,872 features targeting 9532 gene models and the POP2 array 27,648 features targeting 15,789 gene models. All gene models represented on the POP1 array are also present on the POP2 array. See [79] for details of the POP1 array and [77] for details of the POP2 array. For both arrays, see PopulusDB [53] for details of the EST resource used for their production. The merged dataset is available in UPSC-BASE as experiment UMA-9992 and is referred to here as the UPSC-Leaf dataset.

### Populus trichocarpa 'Nisqually-1' Nimblegen microarrays

[80] and [57] make use of an experiment profiling gene expression in different tissues of *Populus trichocarpa *'Niqually-1', the sequenced clone. This experiment has been deposited in GEO (Gene Expression Omnibus, [81]) as series GSE6442. We used only the subset of 16 arrays specific to *P. trichocarpa *'Nisqually-1'. A matrix of normalised expression values was downloaded and gene model means calculated. All subsequent analysis was performed only on probes for nuclear gene models. For OPLS analysis (see below), samples were classed as either leaf or non-leaf. The microarrays used are whole-genome arrays with probes designed to profile 55,794 nuclear and 126 chloroplast and mitochondria gene model sequences in addition to 9,995 unigenes derived from EST sequences [82]. The array platform is in GEO as accession GPL2618. We refer to this dataset as the *P. trichocarpa *Tissue Comparison (Pt-TC) experiment.

### Arabidopsis AtGenExpress

We used the AtGenExpress developmental baseline microarray dataset [56] to visualise the expression of *Arabidopsis *orthologs (defined by best-hit BLAST analysis, [53]) of genes identified in this study. Absolute intensity values were downloaded using the visualisation tool available at AtGenExpress homepage and subsequently visualised using R. The dataset was profiled using the ATH1 Affymetrix expression microarrays which targets 22,746 (> 80%) of known genes.

### Digital Northerns and over-representation anlaysis

Digital Northern heat maps representing the library distribution of ESTs representing gene models within PopulusDB [53,83] were produced using the PopGenIE DigitalNorthern tool [52]. Over-representation of transcription factor families, of transcription factor gene models within leaf libraries, and of miRNAs with predicted target sites within the OPLS leaf genes was tested using the PopGenIE CatFisher tool.

### Transcription factors

2,723 and 2,576 gene models predicted to be transcription factors were downloaded from the public databases Populus Transcription Factor Database [84] and Database of Populus Transcription Factors [85] respectively. The two subsets of transcription factors were merged into a modified single classification scheme by taking the union inside each transcription factor family.

### PCA and OPLS analysis

Principal Component Analysis (PCA) was performed using SIMCA-P (version 11.5, Umetrics, Umeå, Sweden). Normalised M-values were mean-centred and a two-component model was then fitted and visualised.

Orthogonal Projections to Latent Structures (OPLS) was used to model and predict tissue types, treated as different categories (the classes used are described above for the UPSC-TC and Pt-TC experiments). Gene models that were particularly helpful in the discrimination between leaf tissue and non-leaf tissues were subsequently identified using a permutation test essentially according to [29]. All OPLS modelling was performed using R based on in-house produced code. To minimize the problem of over fitting, cross-validation [86,87] was utilised to identify OPLS models with good generalisation properties. The dataset was mean-centred for each gene model prior to modelling. Significant genes were classed as those with FDR-corrected p values < 0.05. P values were generated using the above described permutation test method.

### Clustering and network analysis

Expression information for gene models that were particularly helpful in the discrimination between leaf tissue and non-leaf tissues was extracted from the UPSC-Leaf microarray dataset. The dataset was first filtered to remove microarray slides with >50% missing values followed by a second filter to remove gene models containing >50% missing values, resulting in a final set of 602 arrays. Topological Overlap Matrix (TOM) weighted co-expression was used to construct networks on the filtered dataset and to define transcriptional modules. For a general overview of the method used see [39]. Beta = 6 was used for soft thresholding to fulfil the assumption of a scale-free network (i.e. that some nodes within the network are of greater importance, and will be more highly connected; [88]). Modules of highly interconnected genes were identified using hierarchical clustering and intra-modular connectivity was calculated. Biological function of the individual modules was indicated by testing for over-represented Gene Ontology categories as described below. All analysis was performed in R using scripts based on methods detailed in [39,40] and [41].

### Functional enrichment analysis

Functional over-representation analysis was performed using the BiNGO plugin [89] for the network visualisation software Cytoscape (version 2.5.1, [90]). We used the hypergeometric test and set a Benjamini and Hochberg FDR-adjusted significance level of 0.05 for declaring a GO (Gene Ontology, [51]) category as significantly over-represented. As there is not yet a mature GO release for *Populus*, we have used the best BLAST hit results of EST sequences to *Arabidopsis thaliana *[53] to infer GO using the TAIR (The Arabidopsis Information Resource [91]) 6 release of the *Arabidopsis *genome. Test were performed in the *Arabidopsis *GO-plant-slim ontology. Gene lists identified using OPLS were split into those genes that were highly and lowly expressed in leaves compared to other tissues and the highly expressed gene models were then tested for GO over-representation.

## Abbreviations

QTL: Quantitative Trait Loci; PCA: Principal Component Analysis; OPLS: Orthogonal Projection to Latent Sqaures; TOM: Topological Overlap Matrix [weighted co-expression]; EST: Expressed Sequence Tag; LG: Linkage Group; MIAME: Minimum Information About a Microarray Experiment; TAIR: The Arabidopsis Information Resource; UPSC: Umeå Plant Science Centre; BLAST: Basic Local Alignment and Search Tool; UPSC-TC: UPSC Tissue Comparison; UPSC-LP: UPSC Leaf Primordia; Pt-TC: *Populus trichocarpa *Tissue Comparison; ZF-HD: Zinc Finger Homeobox Domain.

## Authors' contributions

NS drafted the manuscript, analysed the OPLS gene lists and the QTL collocations. AS performed the network and microarray data preparation and analysis and contributed to the manuscript production. MB provided the R scripts and functions for performing the OPLS analysis. SJ supervised the project together with PG and JT. All authors read and approved the manuscript.

## Supplementary Material

Additional file 1 **OPLS leaf genes.** All genes identified as useful for the discrimination of leaf from non-leaf tissues using OPLS. Annotation is based on maximum homology to *Arabidopsis thaliana*. Click here for file

Additional file 2 **OPLS leaf transcription factors.** All genes predicted to be transcription factors and identified as useful for the discrimination of leaf from non-leaf tissues using OPLS. Click here for file

Additional file 3 **OPLS leaf genes containing predicted miRNA binding sites.** All OPLS genes predicted to be targetted by miRNAs. miRNA target predictions are those from [54]. Click here for file

Additional file 4 **Gene ontology biological process categories over-represented in genes predicted to be targetted by more than ten miRNAs.** A functional enrichment analysis was performed on all genes predicted to be targetted by more than ten miRBase [55] ptc-miRNAs. miRNA target predictions are those from [54]. Click here for file

Additional file 5 **Genes within identified transcriptional modules.** Assignment of OPLS leaf genes within transcriptional modules identified using weighted co-expression analysis. Click here for file

Additional file 6 **Overlap between UPSC and nimblegen tissue comparrison leaf genes.** Genes identified using OPLS as informative in discriminating leaf from non-leaf tissues in common to the UPSC-TC and Pt-TC data sets. Click here for file
